# A Skeletal Muscle Ryanodine Receptor Interaction Domain in Triadin

**DOI:** 10.1371/journal.pone.0043817

**Published:** 2012-08-24

**Authors:** Elize Wium, Angela F. Dulhunty, Nicole A. Beard

**Affiliations:** John Curtin School of Medical Research, Australian Capital Territory, Australia; University of Queensland, Australia

## Abstract

Excitation-contraction coupling in skeletal muscle depends, in part, on a functional interaction between the ligand-gated ryanodine receptor (RyR1) and integral membrane protein Trisk 95, localized to the sarcoplasmic reticulum membrane. Various domains on Trisk 95 can associate with RyR1, yet the domain responsible for regulating RyR1 activity has remained elusive. We explored the hypothesis that a luminal Trisk 95 KEKE motif (residues 200–232), known to promote RyR1 binding, may also form the RyR1 activation domain. Peptides corresponding to Trisk 95 residues 200–232 or 200–231 bound to RyR1 and increased the single channel activity of RyR1 by 1.49±0.11-fold and 1.8±0.15-fold respectively, when added to its luminal side. A similar increase in [^3^H]ryanodine binding, which reflects open probability of the channels, was also observed. This RyR1 activation is similar to activation induced by full length Trisk 95. Circular dichroism showed that both peptides were intrinsically disordered, suggesting a defined secondary structure is not necessary to mediate RyR1 activation. These data for the first time demonstrate that Trisk 95′s 200–231 region is responsible for RyR1 activation. Furthermore, it shows that no secondary structure is required to achieve this activation, the Trisk 95 residues themselves are critical for the Trisk 95-RyR1 interaction.

## Introduction

Triadins are a family of proteins consisting of 6 known isoforms, all of which are splice variants of the same gene [1], [2], [3]. Four triadin skeletal (Trisk) isoforms have been identified, namely Trisk 32, Trisk 49, Trisk 51 and the longer 95 kDa Trisk 95 [1], [2], [3], [4], [5]. Two cardiac triadin isoforms are described, the predominantly expressed triadin-1 isoform (which forms a doublet of 35 and 40 kDa) and triadin-3 (75 kDa) [6]. All isoforms share an identical short N-terminal domain and single transmembrane domain, but differ in length in the C-terminal region [6], [7], [8]. Trisk 95 was first identified as a disulfide-linked transmembrane protein located within the junctional sarcoplasmic reticulum (SR) [1], [2], [3], which co-localized with the Ca^2+^ release complex in skeletal muscle. The skeletal Ca^2+^ release complex is a large macromolecular complex, stretching from the surface membrane of the muscle cell, through to the SR, the internal Ca^2+^ store. Central to this complex is a ligand-gated Ca^2+^ release channel, the ryanodine receptor (RyR), which is a 2.2 MDa homotetrameric channel embedded in the SR membrane. The skeletal RyR (RyR1) has recruited the dihydropyridine receptor (DHPR) as a voltage sensor and the skeletal Ca^2+^ binding protein calsequestrin (CSQ1) as a SR Ca^2+^ sensor. RyR1-dependent Ca^2+^ release, initiated by the DHPR response to an action potential, is modified by a variety of factors including ions, co-proteins and the Ca^2+^ environment within the SR lumen. The luminal Ca^2+^ environment is detected and communicated to RyR1 by a complex of proteins found in the SR. CSQ1 is the major Ca^2+^ binding protein which senses and communicates the level of free Ca^2+^ inside the SR to the RyR1 [9], [10], [11]. CSQ’s location close to RyR1 allows such communication, yet CSQ1 does not bind to RyR1 directly. Instead, the CSQ1-RyR1 interaction has been thought to be mediated by Trisk 95 and another structurally similar protein, junctin [12], [13], [14], [15]. Whilst Trisk 95 binds both CSQ1 and RyR1 [15], [16], [17], it does not support the functional CSQ1-RyR1 interaction [13]. Rather, Trisk 95 is deemed to facilitate the orthograde signal between the DHPR and RyR1 in excitation-contraction coupling (EC-coupling) [18]. Results from siRNA-mediated triadin knockdown [19] and pan-triadin-knockout studies [20] support these findings. It should be noted that triadin knockout mice show normal skeletal contractility, which may be due to a contribution from numerous compensatory mechanisms [21].

Various regions on Trisk 95 and junctin have been reported to be responsible for association with RyR1 and CSQ1 in the junctional SR [14], [17], [18], [22]. Trisk 95 association with RyR1 in the SR lumen increases channel activity in lipid bilayers [13]. The binding domain on RyR1 for Trisk 95 has been localized to three residues (D^4878^, D^4907^, and E^4908^) on the M8/M10 luminal loop of RyR1, flanking the pore helix [18], [23]. *In vitro* binding studies [17], [22] have shown that several regions of Trisk 95′s luminal domain can bind to RyR1, with residues 200–232 [17] and 267−280 [22] of the rabbit isoform identified as putative RyR1 binding targets. However, it has remained unclear whether either or both regions are required for RyR1 activation by Trisk 95. Of these Trisk 95 regions, only 200–232 exists in the much shorter cardiac triadin –1 isoform and the residues are homologous between the two isoforms.

Given that luminal addition of Trisk 95 to RyR1 and luminal addition of triadin-1 to RyR2 induce activating responses in the channels [24], [25], it is likely that a region common to both triadin isoforms is responsible for their interactions with RyR1 and RyR2. Thus we have specifically investigated the interaction of Trisk 95 residues 200–232 with RyR1.

The skeletal 200–232 region contains a KEKE motif, which promotes protein association [12], [26], [27], [28]. KEKE motifs are defined as sequences greater than 12 amino acids which lack W, Y, F, P and five positive or negatively charged residues in a row. They must also contain more than 60% K and E/D residues [29]. The Trisk 95 200–232 KEKE motif has been predicted to fold into a β-strand in canine cardiac triadin-1, causing most E and K residues to align and form a polar surface on one side of the strand [26], [27]. The charged side chains of these residues may then form hydrogen bonds with charged residues on protein binding partners, creating a “polar zipper” between the proteins [18], [23], [27]. Curiously in triadin-1, residues found within this KEKE motif have been identified as the binding site for cardiac CSQ (CSQ2; [27]). Given the high sequence homology between CSQ1 and CSQ2, it is possible that CSQ1 also binds to the KEKE motif on Trisk 95 (208–232).

In this manuscript we examine the KEKE motif at residues 200–232 of Trisk 95 and the ability of this region to bind to and regulate RyR1 activity. We find that residues 200–231 of Trisk 95 are able to activate RyR1 in the same way as full length Trisk 95. These results show for the first time that these 31 residues are sufficient to support the regulatory actions of Trisk 95 on RyR1.

## Materials and Methods

### Materials

The monoclonal 34C anti-RyR antibody and monoclonal VIIID12 anti-CSQ1 antibody were from Abcam (Cambridge, MA, USA). Monoclonal anti-triadin (clone IIG12) was from Sigma Aldrich, Inc (Saint Louis, MO, USA), polyclonal anti-junctin antibody was produced against a small N-terminal peptide by IMVS pathology (Adelaide, SA, Australia) and all IgG HRP secondary antibodies were from Santa Cruz (Santa Cruz, CA, USA). Phospholipids were from Avanti Polar Lipids (Alabaster, AL, USA). Streptavidin-agarose was from Thermoscientific (Rockford, IL, USA). SDS PAGE apparatus and consumables were from Bio-Rad (Gladesville, NSW, Australia). All other chemicals were obtained from Sigma-Aldrich (Castle Hill, NSW, Australia).

**Figure 1:**
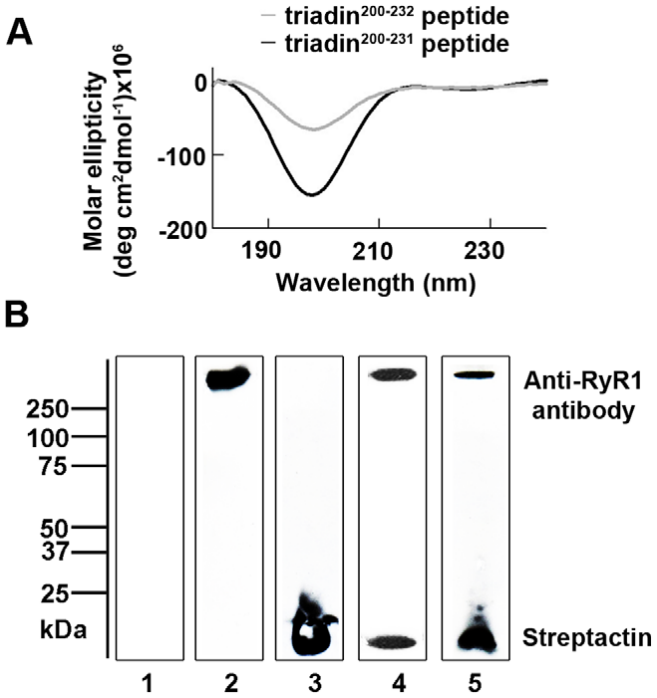
Triadin^200–232^ and triadin^200–231^ peptides bind to RyR1 and exhibit a disordered secondary structure. (A) Circular dichroism spectra averaged from four scans and corrected for the 10 mM sodium phosphate buffer are shown. Both triadin peptides (triadin^200–231^ (black trace) and triadin^200–232^ (grey trace); 0.03 mg/ml) show a negative peak at ∼197 nm, consistent with an intrinsically disordered structure. A positive peak at ∼190 nm and negative peaks at ∼208 and ∼223 nm (indicative of α-helical secondary structure) or a positive peak at ∼195 nm and a negative peak at ∼217 nm (indicative of β-sheet secondary structure), are absent. (B) Western blot, following streptavidin-agarose affinity chromatography, showing the association of RyR1 with biotin tagged triadin peptide. The upper half of the membrane was probed with anti-RyR1 antibody and the lower half was probed with Streptactin-HRP conjugate to identify the biotin tagged peptides. *Lane 1* protein sample eluted from streptavidin-agarose incubated with RyR1 alone; *Lane 2* purified RyR1 alone (control); *Lane 3* biotin tagged triadin^200–231^ peptide alone (control); *Lanes 4 and 5* protein sample eluted from streptavidin-agarose affinity chromatography, where streptavidin-agarose was incubated with biotin tagged triadin^200–232^ or triadin^200–231^ peptide respectively, prior to incubation with RyR1.

### SR Vesicle Isolation and RyR1 Purification

SR vesicles were prepared from back and leg skeletal muscle isolated from New Zealand rabbits [30]. RyRs were solubilized and purified as described previously [31]. The purified RyR1 was concentrated, snap-frozen and stored at −70°C. The protein was resolved on SDS polyacrylamide gels. Following Western Blot, the purity of the solubilized RyR1 fraction was determined by immunoprobing with anti-RyR1, anti-CSQ, anti-triadin and anti-junctin antibodies to detect contamination by these proteins.

**Figure 2:**
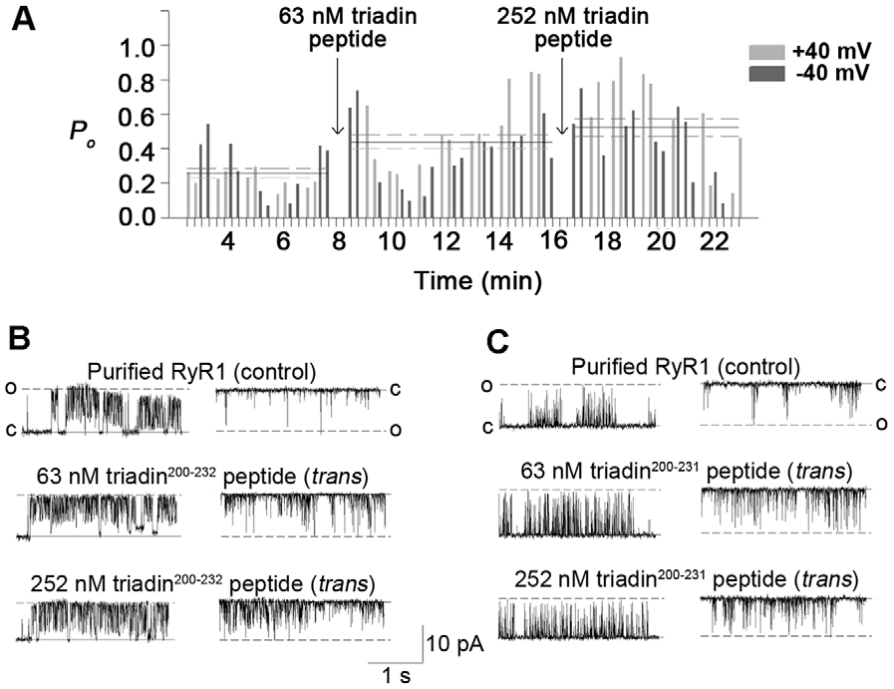
Triadin peptides modulate purified RyR1. (A) Running histogram of a typical bilayer experiment. Open probability (*P_o_*) was measured every 30 s throughout the lifetime of the experiment before and after the addition of 63 nM and then 252 nM triadin^200–231^ peptide (arrows) at +40 mV (light grey bins) and −40 mV (dark grey bins). Data averages for each condition are shown as horizontal broken lines for +40 mV and −40 mV, and median is presented as a horizontal solid line. (B, C) 3 s traces of purified RyR1 channel activity. Activity was recorded at +40 mV (left) where channels are opening upward from zero current (c, continuous line) to maximum open conductance (o, broken line) and at −40 mV (right), where channel openings are downwards from zero current (c, continuous line) to maximum open conductance (o, broken line). *Top panel –* control recording of purified RyR1 prior to the addition of triadin peptide; *middle and bottom panel* – after the addition of 63 nM and 252 nM triadin peptide to the *trans* chamber. (B) Shows addition of triadin^200–232^ peptide, and (C) shows addition of triadin^200–231^. Control *P_o_* in the absence of peptide was 0.46±0.12 at +40 mV and 0.38±0.17 at −40 mV (for triadin^200–232^), and 0.14±0.07 at +40 mV and 0.29±0.17 at −40 mV (for triadin^200–231^).

**Figure 3:**
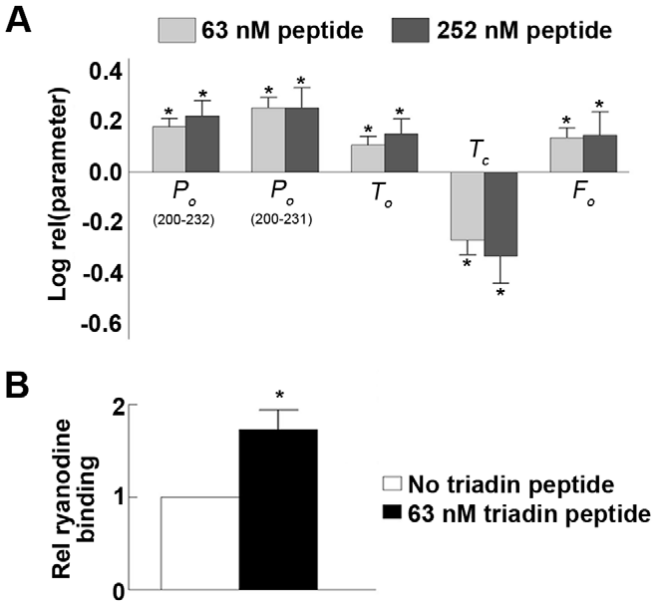
Triadin peptide modulates purified RyR1 single channel open time, closed time and closed frequency. (A) Average data for open probability (*P_o_*) in presence of triadin^200–232^ and triadin^200–231^ (n = 10), data from both peptides combined (see results text, collectively termed triadin peptide, n = 11–20) for each of the following parameters; open time (*T_o_*), close time (*T_c_*) and open frequency (*F_o_*), collected at −40 mV and +40 mV. All data is expressed as relative mean data (Log rel (parameter)). Relative mean *P_o_* (log rel *P_o_*) is the average of differences between the log_10_ of the *P_o_* in the presence of either 63 nM or 252 nM triadin peptide (log_10_ *P_o_*_Pep_) and log_10_ of the control *P_o_* (log_10_ *P_o_*_Con_) for each channel. Log rel *T_o_* is log_10_ *T_o_*_Pep_-log_10_ *T_o_*_Con_, log rel *T_c_* is log_10_ *T_c_*_Pep_-log_10_ *T_c_*_Con_ and log rel *F_o_* is log_10_ *F_o_*_Pep_-log_10_ *F_o_*_Con_. (B) [^3^H]ryanodine binding to purified RyR1 in the absence and presence of 63 nM of triadin peptide. [^3^H]ryanodine binding is measured as pmol ryanodine/mg RyR1. Data is expressed relative to binding recorded in the absence of peptides (rel ryanodine binding). Significance (p≤0.05) is indicated for each concentration compared to activity recorded prior to addition of peptide (*).

### RyR1 ΔM_1,2,3_ Mutant Protein Expression

HEK-293T cells were transfected with a mammalian expression vector, pCIneo, encoding the GFP tagged RyR1 ΔM_1,2,3_ mutant using calcium phosphate precipitation. RyR1 ΔM_1,2,3_ contains three alanine substitution mutations; D4878A, D4907A and E4908A and was kindly provided by Professor R. Dirksen [18]. Forty eight hours post-transfection cells were homogenized in *homogenizing buffer* (300 mM sucrose, 5 mM imidazole, pH 7.4) using an IKA Ultra-Turrax T10 homogeniser (IKA Works (Asia) Sdn Bhd, Selangor, Malaysia) before centrifuging at 75130× g for 2 h at 4°C. The resultant pellet was further homogenized manually in *homogenizing buffer* using a glass homogenizer and the presence and purity of RyR1 ΔM_1,2,3_ was assessed using SDS PAGE and Western Blot.

### Electrophoresis and Western Blot

SDS-PAGE and Western Blot are described in [32].

### Peptide Synthesis

Peptides corresponding to the 200–232 and 200–231 residue sequence of Trisk 95 [18] were obtained from the Biomolecular Resource Facility, The John Curtin School of Medical Research (Canberra, ACT, Australia). Peptides were synthesized using the 9-fluorenylmethyloxycarbonyl method on a CEM Microwave-assisted Peptide Synthesizer (CEM Corporation; Matthews, NC, USA) and purified by one round of C18 reversed-phase HPLC. As required, the N-terminus was protected by acetylation. The RyR1^4871–4910^ peptide, containing the putative Trisk 95 binding domain on the RyR1 [18], was produced and HPLC purified by GL Biochem (Shanghai) Ltd (Shanghai, China). Peptide purity was confirmed using mass spectroscopy. The peptide sequences are as follows:

Triadin^200–232^ peptide:^ 200^KTVTKEEKKARTKEKIEEKTKKEVKGVKQEKVK^232^.

Triadin^200–231^ peptide:^ 200^KTVTKEEKKARTKEKIEEKTKKEVKGVKQEKV^231^.

RyR1^4871–4910^ peptide:^ 4871^EPDMKCDDMMTCYLFHMYVGVRAGGGIGDEIEDPAGDEYE^4910^.

For affinity chromatography a biotin tag was conjugated to the N-terminal end of the triadin^200–232^ and triadin^200–231^ peptides. A linker sequence of 4 residues (KPET), corresponding to residues 196–199 of rabbit skeletal Trisk 95, was included between the biotin tag and peptide sequence to minimize the risk of the biotin tag interfering with the binding domain for the RyR1.

### Circular Dichroism

Circular Dichroism (CD) spectra of the peptides were obtained using a Chirascan spectrometer (Applied Photophysics, Surrey, UK) held at 20°C by a Melcor Peltier temperature controller. Peptides were diluted to 0.03 mg/ml in a buffer composed of 10 mM di-sodium hydrogen orthophosphate dihydrate and 10 mM sodium dihydrogen orthophosphate monohydrate (pH 8.0), and spectra were recorded between 180 nm and 240 nm in a 0.1 cm path length cell. Each spectrum was averaged from 4 scans and corrected for buffer absorbance.

### Streptavidin-agarose Affinity Chromatography

Briefly, biotinylated peptides (2 mg/ml in *affinity buffer* containing 150 mM NaCl, 20 mM MOPS and 1 mM CaCl_2_ at pH 7.4) were coupled to 200 µl streptavidin-agarose and incubated by rotation for 3 h at room temperature or 4°C overnight. Unbound peptide was removed by centrifugation and the streptavidin-agarose-peptide slurry was washed 5 times with 5× bed volume of *affinity buffer*. Purified RyR1 (25 µl of 1 mg/ml in *affinity buffer*) was pre-cleared in streptavidin-agarose with rotation overnight at 4°C, to eliminate non-specific binding, and the supernatant (pre-cleared RyR1) was incubated with the streptavidin-agarose-peptide slurry with rotation overnight at 4°C. The streptavidin-agarose-peptide-RyR1 slurry was washed in *affinity buffer* as above. Proteins and peptides were eluted from the streptavidin-agarose by boiling the samples in *sample buffer* (200 mM Tris HCl (pH 6.8), 20% β-mercaptoethanol, 40% glycerol, 8% SDS, and 0.08% bromophenol blue) for 5 min. Proteins and peptides were identified by immunoprobing with anti-RyR antibody and streptactin (to detect biotinylated peptides) after SDS PAGE and Western Blot.

### [^3^H]ryanodine Binding

[^3^H]ryanodine binding was performed as described in [18], except that 5 µg of purified RyR1 and 63 nM of triadin peptide were used. In experiments where triadin was pre-incubated with RyR1^4871–4910^ peptide, 630 nM RyR1^4871–4910^ peptide was used to saturate triadin. [^3^H]ryanodine binds to the RyR preferentially when it is in its open state and binding is an indicator of RyR1 activity [33].

### Single Channel Recording and Analysis

Artificial planar bilayers separating two baths (*cis* and *trans*) were formed as previously described [9], [32]. Purified RyR1s (3–4.5 µg), SR vesicles (∼50 µg) or expressed RyR1 ΔM_1,2,3_ (3–4.5 µg) were added to the *cis* solution so that the cytoplasmic surface of RyR1 faced the *cis* solution after incorporation into the lipid bilayer [9]. The composition of the incorporation solutions were as follows: *cis*: 230 mM CsMS, 20 mM CsCl, 1 mM CaCl_2_, and 10 mM TES (pH 7.4); and *trans*: 30 mM CsMS, 20 mM CsCl, 1 mM CaCl_2_, and 10 mM TES (pH 7.4). After incorporation of a channel, *trans* [Cs^+^] was raised from 50 to 250 mM with the addition of 200 mM CsMS and the *cis* solution was modified by the addition of 2 mM ATP and ∼4.5 mM BAPTA to achieve a final free [Ca^2+^] of 100 nM, determined using a Ca^2+^ electrode (Radiometer Analytical SAS, Villeurbanne, France). Peptides were added to the *trans* chamber. Orientation of the incorporated RyR1 was confirmed by characteristic responses to changes in cytoplasmic [Ca^2+^], ATP and ruthenium red [34], [35]. The data was filtered at 1 kHz and sampled at 5 kHz.

Single channel parameters from 90–180 s of recording were obtained using the Channel 2 program (developed by P.W. Gage and M. Smith, John Curtin School of Medical Research, Canberra, Australia). The threshold levels for channel opening were set to exclude baseline noise at ∼20% of the maximum single-channel conductance, and open probability (*P_o_*), mean open time (*T_o_*), mean closed time (*T_c_*) and open frequency (*F_o_*) measured. All electrical potentials are expressed as that of cytoplasmic solution relative to the luminal solution. Single channel recordings were obtained at +40 mV and −40 mV. These voltages were chosen to ensure a large current flow through the channel, which would maximize the signal to noise ratio, without easily breaking the bilayer. Currents at both positive and negative potentials were monitored to assess possible voltage dependent effects of the peptide. Measurements were carried out at 23±2°C.

### Statistics

Average data are presented as mean ± SEM. To reduce the effects of variability in control parameters (*P_o_*_Con_, *T_o_*_Con_, *T_c_*_Con_, *F_o_*_Con_), and to evaluate parameters after peptide addition (*P_o_*_Pep_, *T_o_*_Pep_, *T_c_*_Pep_, *F_o_*_Pep_), data were expressed as the difference between log_10_X_Pep_ and log_10_X_Con_ for each channel (e.g., log_10_ *P_o_*_Pep_ − log_10_ *P_o_*_Con_). The difference from control was assessed with a paired *t*-test. A P value of <0.05 was considered significant.

**Table 1:** Channel open probability parameter values for native and purified RyR1 measured in the presence and absence of 63 nM Triadin peptide and 63 nM full length Trisk 95.

	*P_o_*							
RyR1	− triadin peptide	+ triadin peptide	Fold change	n	−Trisk 95	+ Trisk 95	Fold change	n
**Native**	0.771±0.054	0.856±0.053	1.12	6	–	–	–	–
**Purified**	0.273±0.057	0.402±0.074*	1.68*	20	*0.111±0.017*	0.207±0.02*	1.86*	*40*

The average open probability (*P_o_*) values calculated from lipid bilayer experiments are shown. *P_o_* for each channel type and presence or absence of peptide/Trisk 95 is measured from 90 s of activity at both +40 mV and −40 mV (pooled). The fold change is the *P_o_* value after *trans* addition of triadin peptide or full length Trisk 95, relative to *P_o_* in the absence of triadin peptide/Trisk 95. Trisk 95 was isolated from rabbit skeletal muscle and these data (*in italics*) have been previously published in [13] and are included here for comparison. Significant (p≤0.05) differences in *P_o_* upon addition of triadin peptide or Trisk 95 are indicated for each condition compared to its control (*).

**Figure 4:**
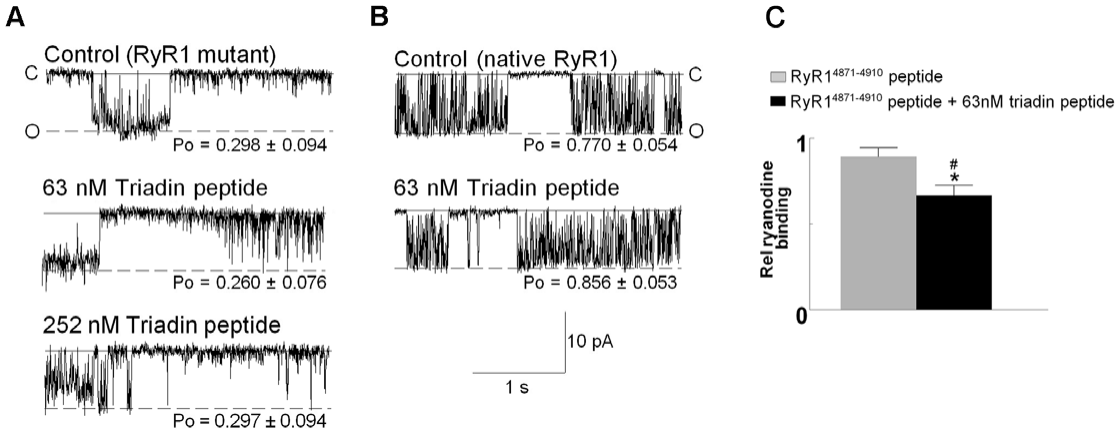
Triadin peptide does not activate mutant or native RyR1, or when its RyR1 binding site is blocked. (A–B) 3 s traces of RyR1 channel activity at −40 mV. Channels are opening downwards from zero current (c, continuous line) to maximum open conductance (o, broken line). (A) RyR1 ΔM_1,2,3_ mutants (with Trisk 95 binding residues mutated) in absence of triadin peptide and in the presence of *trans* 63 nM and 252 nM peptide. (B) Native RyR1 in the absence and presence of *trans* 63 nM triadin peptide. Average open probability (*P_o_*) of data, collected at −40 mV and +40 mV (n = 6–10) is displayed below each trace. (C) [^3^H]ryanodine binding to purified RyR with triadin binding blocking peptide (RyR1^4871–4910^ ) in the absence and presence of 63 nM triadin peptide. [^3^H]ryanodine binding is measured as pmol ryanodine/mg RyR1 and data is expressed relative to binding recorded in the absence of either peptide (rel ryanodine binding). All values are presented as relative to control. Asterisks (*) indicate a significant difference (p≤0.05) in binding from RyR1 control (no peptide); crosshatch (#) indicates a significant difference (p≤0.05) in binding from RyR1 in the presence of RyR1^4871–4910^.

## Results

### Secondary Structure of Triadin Peptides

Two peptides were used to assess the Trisk 95-RyR1 interaction. One peptide (triadin^200–232^) contains a canonical KEKE motif with 60.6% K/E/D residues. The other peptide (triadin^200–231^) is missing the final residue of triadin^200–232^ which technically puts it outside the definition of a KEKE motif (it only contains 57.6% K/E/D). Both peptides were assessed for their secondary structure.

Kobayashi et al. [27] proposed that residues 210–224 of a canine cardiac triadin-1 KEKE motif form a β strand to allow triadin-1 to interact with CSQ2. The same KEKE motif is found within residues 200–232 of Trisk 95 and may also employ a β strand structure to bind the RyR1. If this is the case, the triadin^200–232^ peptide may adopt a β strand structure and self aggregate to display β-sheet secondary structure, as such structures have been observed with peptides as short as 13 residues in length [36], [37]. Therefore, we investigated the structural profile of the triadin^200–231^ and triadin^200–232^ peptides using circular dichroism (CD). The CD spectra revealed that the secondary structure of both peptides was disordered (Fig. 1A). Neither spectra display peaks characteristic of either α-helical or β-sheet structure. Disrupting the KEKE motif by shortening the peptide (to triadin^200–231^) did not significantly alter its structure. The sequence of the two peptides was identical apart from the lack of residue 232 in the truncated peptide. Thus, not unexpectedly, the loss of the classically defined KEKE motif, did not affect the peptide’s secondary structure.

### Triadin Peptide Association with RyR1

The ability of the peptides to associate with RyR1 was considered next. Biotinylated triadin^200–232^ or triadin^200–231^ peptide was coupled to immobilized streptavidin-agarose prior to incubation with purified RyR1. The resultant peptide-protein complex was eluted as described (Methods), and identified post Western Blot by immunoprobing with anti-RyR1 and streptactin (biotin probe). There was a substantial association between the purified RyR1 and both the triadin^200–232^ and triadin^200–231^ peptides (Fig. 1B; lanes 4 and 5). No detectable RyR1 bound to the streptavidin-agarose when the biotinylated triadin peptide was absent (Fig. 1B; lane 1). Therefore, both peptides bound RyR1 despite being primarily unstructured, and they did so to the same extent as full length Trisk 95 under the same conditions [18].

### Regulation of Purified RyR Channel Activity by Triadin Peptides

It is not known which RyR1 binding regions of Trisk 95 [17], [22] are responsible for the protein’s robust activation of RyR1. To test our hypothesis that residues 200–232 in Trisk 95 contribute to RyR1 modulation, we examined the effects of the triadin^200–232^ and triadin^200–231^ peptides on purified RyR1 activity using the single channel lipid bilayer technique. Purified RyR1 channels were incorporated into lipid bilayers in the presence of 100 nM *cis* Ca^2+^ and 1 mM *trans* Ca^2+^. After baseline RyR1 activity was recorded, the peptides were added at a concentration of 63 nM to the *trans* chamber, which is equivalent to the molar concentration of full length Trisk 95 and cardiac triadin-1 used in previous lipid bilayer studies [24], [25]. To determine whether this was a saturating concentration of peptide, the RyR1 response to a 4-fold greater peptide concentration (252 nM) was also tested.

Addition of either triadin^200–232^ or triadin^200–231^ peptide resulted in a significant increase in RyR1 activity. This activation was sustained over the lifetime of the bilayer, as demonstrated by measuring open probability (*P_o_*) over the course of the experiment (Fig. 2A). There was no significant difference between the *P_o_* increase induced by the two peptides (p = 0.132, Fig. 2B & C, 3A). The triadin^200–232^ peptide caused a 1.49±0.11-fold increase in *P_o_* (in the presence of peptide relative to absence of peptide), while triadin^200–231^ caused a 1.8±0.15-fold increase. There was no significant difference in the degree of activation evoked by either peptide at −40 mV and +40 mV (Fig. 2B), or at 63 nM and 252 nM (Fig. 2, 3A). Thus maximal RyR1 activation was achieved by 63 nM peptide. As both peptides mediated similar RyR1 activation, data from the two peptides is combined and the peptides collectively referred to as triadin peptide in all subsequent experiments. Combined lipid bilayer data demonstrated the triadin peptide increases *P_o_* via a significant abbreviation of channel closed time, and significant increases in both the open time and the frequency of openings (Fig. 3A). Results obtained using [^3^H]ryanodine binding supported the single channel data. The addition of triadin peptide to purified RyR1 evoked a significant 1.73±0.21-fold increase in [^3^H]ryanodine binding (Fig. 3B), which indicates increased channel activity and is consistent with the single channel data.

Comparison of the regulatory effects of full length Trisk 95 (which was isolated and purified from rabbit skeletal muscle) and the triadin peptide reveals that both caused a similar increase in *P_o_* (Table 1) and increase in [^3^H]ryanodine binding [18]. Therefore, the triadin peptide (specifically residues 200–231) replicates the effect of Trisk 95 on RyR1. It is interesting to note that any post-translational modification (such as disulfide formation and glycosylation) of Trisk 95 from rabbit skeletal muscle is not present in the triadin peptide and therefore appears unimportant for RyR1 activation by Trisk 95.

### Triadin Peptide Regulation of RyR1 with Mutated or Occupied Trisk 95 Binding Site

Three residues of RyR1 (D^4878^, D^4907^, and E^4908^) are critical for the protein’s ability to interact with Trisk 95. When these RyR1 residues are mutated to alanines, Trisk 95 no longer physically or functionally interacts with RyR1 [18], [23]. In a control experiment, the RyR1 mutant construct (RyR1 ΔM_1,2,3_) containing the three critical mutations (D4878A, D4907A and E4908A) was incorporated into a lipid bilayer. There was no significant change in channel activity after addition of either 63 nM or 252 nM *trans* triadin peptide (Fig. 4A). The inability of triadin peptide to activate the RyR1 ΔM_1,2,3_ mutant indicates that it requires the same residues as the Trisk 95 protein to bind to and activate RyR1.

A similar conclusion was reached when triadin peptide was added to native RyR1 which retains endogenous Trisk 95 (as well as junctin and CSQ1). SR vesicles (containing native RyR1) were incorporated into lipid bilayers and 63 nM triadin peptide was added to the *trans* chamber (Fig. 4B). Triadin peptide did not activate RyR1 as strongly in the presence of endogenous Trisk 95 as in its absence (compare Fig. 2B and 4B). The fact that triadin peptide did not produce the same degree of RyR1 activation when endogenous Trisk 95 is present, is consistent with the peptide associating with the same site as Trisk 95 on the RyR1. This, together with the data from the RyR1 ΔM_1,2,3_ mutant, provides strong evidence that residues 200–231 of Trisk 95 replicates all the nuances of RyR1 regulation displayed by the full length protein [13].

### Blocking the RyR1 Binding Site on Trisk 95

We examined the effect of blocking the RyR1 binding site on triadin using a RyR1 peptide (with the sequence of the triadin binding site and surrounding residues) on [^3^H]ryanodine binding. Triadin peptide was pre-incubated with a 10x greater concentration of RyR1^4871–4910^ peptide, prior to addition to purified RyR1. The RyR1^4871–4910^ peptide should saturate all residues on the triadin peptide which interact with RyR1 and we expected to see no additional change in [^3^H]ryanodine binding when the combined triadin peptide plus RyR1^4871–4910^ was added to purified RyR1. RyR1^4871–4910^ alone did not influence [^3^H]ryanodine binding (Fig. 4C). Curiously, we found triadin peptide in fact reduced [^3^H]ryanodine binding when pre-incubated with RyR1^4871–4910^, indicating RyR1 inhibition (Fig. 4C). This inhibition could be due to residues within the triadin peptide that did not interact with RyR1^4871–4910^ (i.e. not involved in the specific Trisk 95-RyR1 interaction) binding to RyR1. This binding may have been to luminal or cytoplasmic domains of RyR1 as peptides can access both the cytoplasmic and luminal faces of RyR1 in the [^3^H]ryanodine binding assay. In lipid bilayers, the peptides can only access the luminal face of RyR1, and there was no inhibitory effect on the channel when the Trisk 95 binding sites on RyR1 was mutated or occupied by endogenous Trisk 95 (Fig. 4A, B). Taken together, these results show that the triadin peptide inhibits RyR1 only when (i) the luminal RyR1 binding site of the peptide is unavailable and (ii) when sites on the cytoplasmic side of the RyR1 are exposed. Blocking the critical RyR1 binding residues on the triadin peptide prevented the stimulating effect of the triadin peptide on RyR1 (Fig. 4C), consistent with Trisk 95 residues 200–231 forming the activating domain for RyR1.

## Discussion

In this study we show that two Trisk 95 peptides, corresponding to residues 200–232 (triadin^200–232^) and 200–231 (triadin^200–231^), bind to and activate RyR1. The truncated peptide did not lose the ability to regulate RyR1 despite no longer containing a true KEKE motif. Both peptides significantly increased RyR1 *P_o_* in lipid bilayer studies, and the same is inferred from [^3^H]ryanodine binding data. The degree of RyR1 activation caused by the peptides replicates that by full length Trisk 95 under identical experimental conditions [13], [18]. The peptides do not activate RyR1 when their Trisk 95 binding site is mutated or occupied. Our findings indicate that both triadin peptides are intrinsically disordered, and suggest that neither a rigid secondary structure nor an artificially defined KEKE motif determine the peptides’ ability to bind to and activate RyR1. Kobayashi et al [27] hypothesized that specific residues and structure are important for maintaining the triadin-1/CSQ2 interaction. Our results indicate that structure is not important and that specific residues between 200–231 are responsible for binding to RyR1 and activating the channel.

As Trisk 95 residues 200–231 are sufficient to mimic the activation of RyR1 by full length Trisk 95, it seems unlikely that the other luminal region on Trisk 95 shown to bind the RyR1 with high affinity (residues 267–279; [22]) contributes to the functional effects of the RyR1-Trisk 95 association. In addition, the hydrophobic nature of Trisk 95 residues 267–279 suggests they are unlikely to form binding partners for the acidic D^4878^, D^4907^ and E^4908^ residues on RyR1, whereas the basic residues of Trisk 95 200–231 would provide appropriate binding partners. That said, we cannot exclude the possibility that residues 267–279 help stabilize the Trisk 95-RyR1 interaction *in vivo*. In fact, Trisk 95 contains a cysteine at residue 270. Earlier studies suggest that under certain circumstances C^270^ may form a disulfide bond with RyR1 (or other SR proteins) providing a stabilizing thiol link between the two proteins [38]. Indeed, reducing agents are routinely used in our lab to dissociate Trisk 95 from RyR1.

Current dogma holds that in skeletal muscle, Trisk 95 and junctin remain closely and simultaneously associated with both CSQ1 and the RyR1. However, junctin alone supports the ability of CSQ1 to inhibit RyR1 activity [13]. Trisk 95 does not mediate the functional coupling between RyR1 and CSQ1. *In vitro* studies of purified proteins indicate that Trisk 95 binds to RyR1 and to CSQ1 individually [14], [16], [17], [18], but simultaneous binding of CSQ1 and RyR1 to Trisk 95 has not been demonstrated. We have shown that residues within 200–231 are critical for Trisk 95 association with, and its functional influence on, RyR1. Based on the CSQ2-triadin-1 association in canine cardiac muscle [27], it is predicted that CSQ1 would bind to Trisk 95 residues 218–232 in rabbit. Therefore, the binding sites on Trisk 95 for RyR1 and CSQ1 may overlap such that both proteins would be unlikely to associate with Trisk 95 simultaneously. This could explain why CSQ1 induced RyR1 inhibition is mediated by junctin alone [13].

If Trisk 95 does not bind to the RyR1 and to CSQ1 simultaneously, it is tempting to speculate that domain swapping between CSQ1 and RyR1 for the binding site on Trisk 95 may contribute to the modulation of SR Ca^2+^ release during EC coupling. The affinity between CSQ1 and Trisk 95 becomes lower as the [Ca^2+^] increases [12], [14]. So, when the SR store [Ca^2+^] is high (i.e. in resting muscle fibres), Trisk 95 may preferentially bind to RyR1 (and not CSQ1), activating the channel to facilitate Ca^2+^ release. The drop in SR free Ca^2+^ during contraction would promote Trisk 95 association with CSQ1, relieving Trisk 95 activation of RyR1 and thus conserving the store Ca^2+^ load and reducing SR Ca^2+^ leak. This hypothesis is consistent with the role suggested for Trisk 95 in EC-coupling, [18], whereby the luminal Trisk 95-RyR1 interaction activates the RyR1 channel in response to the depolarization-evoked signal from the DHPR.

We have provided novel evidence that residues 200–231 of Trisk 95 mediate the RyR1 activation by full length Trisk 95. Furthermore, we found that these residues were important despite the lack of a β-strand or helical secondary structure. Therefore the residues within 200–231 of Trisk 95, and not their specific structure, are responsible for RyR1 activation by full length Trisk 95. It seems plausible that basic residues within 200–231 will form binding partners for D^4878^, D^4907^ and E^4908^ of RyR1. Future experiments will be performed to identify which individual residues between Trisk 95 200–231 are responsible for binding to RyR1 and activating the channel.
